# The New York Institute of Stomatology

**Published:** 1898-08

**Authors:** 

**Affiliations:** The New York Institute of Stomatology


### THE NEW YORK INSTITUTE OF STOMATOLOGY.

A REGULAR meeting of the Institute was held Tuesday evening, April 5, 1898, at the residence of Dr. J. Stedman Converse, 42 West Fortieth Street, the President, Dr. E. A. Bogue, in the chair.

*The President.*—Our fellow-member, Dr. George A. Maxfield, of Holyoke, Massachusetts, is present, and will address us on the subject of "Hypercementosis."

*Dr. Maxfield.*—Mr. President and gentlemen, I have not much that is new, but I have thought it might interest some to see the specimens I have in illustration of the subject. I have one case with a complete history from the beginning, and there are two other cases with results that will be of interest. The literature on the subject is very limited, and I was very much disappointed when taking up the study to find so little. The best that has ever been written on the subject is by Professor Guilford, of Philadelphia, in the "American System of Dentistry." There is one point that seems to have been made by all the writers, that hardly any trouble is experienced from hypercementosis except where there is a large deposit of cementum. My experience has been that the trouble comes from the small deposits. Here is a case—the first right inferior molar—in which I am confident I was wholly the cause of the trouble. This patient came to me when I was in college at Philadelphia, and I placed two large gold fillings in each approximal surface of the tooth. I used Dr. Bonwill's mechanical mallet, and probably, as most students do, used a heavy blow. I never had that feature brought to my attention as it was in our State examinations at Boston last month. I noticed a number of the candidates who were using extremely heavy blows with their automatic mallets. In this case trouble commenced about three months after the tooth was filled. I commenced treatment by making external applications to the gums, then removed one of the approximal fillings, and finally destroyed the pulp and removed it. As the pain still continued after three or four months, I extracted the tooth, and there was the hypercementosis on each root. I am very confident that the irritation caused by heavy malleting was the cause of that deposit.

I have here—Card No. 3—a case of a second bicuspid. This man had a great deal of neuralgia, which commenced several months before he came to me. It would come on in paroxysms, last for several hours, and then stop. At such times he could not tell which tooth was troubling, but the pain was on that side of the face. After studying his mouth I removed this tooth and the trouble ceased.

Another case (I pass around casts made before and after the tooth was extracted). This man came in, having suffered for nine years with severe neuralgia through the front and right side of the face. He wanted the lower incisors removed; it was in the winter, and the cold air caused severe pain in these teeth. After looking over his mouth, I told him I would take a cast of the mouth and study the case. When he came again I removed the first right inferior bicuspid, and there has been no recurrence of the neuralgia.

The lady from whom this cast was made suffered a great deal with neuralgia on the left side of the face, and complained of pain in the ear. At that time I was not able to diagnose the trouble, and did not until a few years ago. One can see that all the trouble must have come from that superior first molar, which had dropped down through loss of its antagonist, and there must have been hypercementosis. The neuralgia and the condition of this superior molar are typical symptoms for the diagnosis of this trouble.

I have here a specimen, an inferior left third molar. This man had been a sufferer from neuralgia for seven years, and had been treated by physicians without effect. After examining the mouth I determined at once what the trouble was and removed the tooth, and his neuralgia ceased. In this case the tooth, instead of standing upright, tipped forward, pressing against the second molar.

Here is a specimen showing erosion, which is very distinct indeed. Undoubtedly this was the cause of the hypercementosis.

Hypercementosis does not always cause trouble. I have a case here of a tooth that I extracted February 25, in which I could not determine from the pain that there was any hypercementosis. As all the other teeth back of the first bicuspid were decayed down to the gum, I removed this tooth because I had to put in a plate, and I did not want to have it in the way.

I have here a specimen upon which the deposit was evidently caused by excessive function. On the first cast it may be noticed that every tooth except the one I extracted has an antagonist.

Some nine years ago a travelling quack came to Holyoke to extract teeth. He operated on the young man represented by this cast, who was then about seventeen years old. The lower left cuspid projected against the lip, was very hard to extract, and his jaw was injured. About three months after neuralgia set in, and remained constant from that time. Now, taking into account the injury from the extraction and that the tooth had no antagonist, I determined that this tooth had hypercementosis and removed it. The specimen shows the diagnosis was correct.

The tooth that is being passed around on Card No. 3 had an antagonist until a few years ago, when I removed the bicuspid above it. The man had had quite a nervous breakdown in the mean time, and after looking his mouth over, noticing that was the only tooth that did not have an antagonist, I determined that was the tooth which caused the trouble. Extraction showed it was the one. One thing I would like to speak of, and that is diagnosing by the bunch through the gum. I have never yet felt one, and in all the cases of trouble I have had there were not excessive deposits. I cannot describe the pain except as a pulp pain and one the patient cannot locate. To diagnose the trouble is something intuitive. When the patient describes the pain one seems to realize at once whether it comes from the pulp, for in none of these cases did I find any soreness on tapping the tooth. If a patient complains of pain in the ear, as a rule we can be sure that a tooth causes the pain. In all these cases but the first one the patients complained of earache.

*Dr. S. E. Davenport.*—I would like to ask Dr. Maxfield whether every tooth, so far as he can recollect, which has been proved in his experience to be in this condition, was without an antagonist.

*Dr. Maxfield.*—Whenever I have found this pain there has been no antagonist except in the first case, in which, as I have explained,

I was myself the cause of the hypercementosis. The lack of an antagonist is of much signification. In these cases more pain results than where the hypercementosis results from excessive function.

*Dr. F. Milton Smith.*—I would ask whether these teeth were loosened or firm.

*Dr. Maxfield.*—Very firm, indeed.

*Dr. S. H. McNaughton.*—Were the pulps alive or dead in most of these teeth, and were any of the living teeth treated by killing the pulp?

*Dr. Maxfield.*—Only in one case,—the first card I passed around. In all cases of pain resulting from hypercementosis treatment is of no avail. The tooth must be extracted.

*The President.*—Time does not permit us to continue the discussion of this subject. Dr. Ewing is with us to review the results of some recent investigations on the histology of dentine, and we will now ask him to do so.

*Dr. James Ewing.*—I have accepted the invitation to say a few words concerning a recent article relating to the minute structure of dentine. The article that I refer to is one by a German investigator—Morgenstern—which appeared in the *Archiv für Anatomie und physiologische Anatomie*, Abt., 1896, p. 326. It refers to an apparently successful effort to demonstrate the existence of non-medullated nerve-fibres in the dentinal tubule. This has been a mooted point for many years, but Morgenstern claims in this article that these structures have been successfully demonstrated by the use of a slight modification of Golgi's method invented by Ramón y Cajal. The conclusion reached is the result of a long series of experiments, extending over a period of six or eight years, which Morgenstern had undertaken in order to complete his demonstration.

The first point which I want to bring out is the method which he uses. He says it is a modification of Golgi's method, this modification consisting in the use of osmic and chromic acids as a fixing fluid. It is not necessary to go fully into the modification now, as it would require the description of a number of fluids, but it is enough to say that he proceeded as follows: He took the fresh dentine, chipping it off in small pieces, and in some cases sawing some masses from the main bulk of the tooth. These he placed in a solution of chromic and osmic acids, and after remaining in this solution for a week or ten days, the fluid being frequently renewed, they were removed and treated in alcohol, and finally by silver salts, and at the end of three or four months the sections were ground down and examined. So much for the method which he uses.

The second point is what he found. He found that the differentiation of the structures in the dentinal tubules was very much clearer than he had secured by other methods. He recognized the dentinal fibres very much more distinctly, and in addition he found structures resembling the ordinary non-medullated nerve-fibres,—that is, they came out as black lines encircling the dental fibres and showing the characteristic nodal thickenings. In addition to these fibres, he mentions a point which seems to me of equal importance almost; that is, the presence of a set of nuclei in the dentinal tubules which apparently belong to endothelial cells. In these two points are the chief results of Morgenstern's work. He demonstrates the fine black lines with nodal thickenings which correspond exactly with non-medullated nerve-fibres, and he finds this separate set of nuclei, probably of endothelial cells. He goes into considerable detail in following the course of these fibres. They start at the bases of the dentinal tubules and run through the lateral anastomoses of these tubules. They run for the most part in the dentinal tubules, but also in the intertubular substance. Unfortunately the precipitation of metallic salt in most tissues is an uncertain method of demonstrating tissue structures, for the reason that there is not always a chemical union between the metallic salt and the structures, the salt precipitating sometimes in the spaces and sometimes in the structures. The question is whether these delicate lines which Morgenstern demonstrates are actual structures or whether they are spaces. The weight of evidence is certainly in favor of the belief that Morgenstern has demonstrated nerve-fibres, because where the salts have been deposited in the spaces, and not in the structures, the spaces have been very irregular in outline. The exact termination of these fibres he has not worked out, but he has demonstrated a very rich anastomosis between them; in fact, to a large extent, he has apparently solved the question of the nerve-supply of dentine. The presence of such nerve-fibre in dentinal tubules would, of course, explain the marked sensitiveness of dentine, which is a matter of common clinical observation.

It has appeared to me to be a very important fact, as stated, that he has found evidence to show that the fibres run in lymphatic spaces lined by endothelial cells.

I am reminded here of the specimens of Dr. Allan's, prepared by Miller, showing the growth of bacteria in the dentinal tubules. On first seeing these I was astounded at the brilliancy of the demonstration. It is much easier to understand the deep penetration of these bacteria when it appears that the dentinal tubules are lined by endothelial cells, as bacteria readily penetrate lymphatic spaces.

The whole result of Morgenstern's work is certainly a just reward of a careful series of painstaking and honest studies in this interesting subject.

*The President.*—Will Dr. Ewing kindly inform us whether Morgenstern's paper has been translated into English?

*Dr Ewing.*—No, sir. It can be found at the Academy of Medicine, in the *Archiv für Anatomie und physiologische Anatomie*, 1896, page 326. There are two parts of that journal, and this is in the anatomical part.

The President then introduced Professor J. L. Wortman, of the American Museum of Natural History, who delivered an address upon "The Relationship of Dental Organs to the Doctrine of Evolution," illustrated by lantern slides.

This important paper will appear as soon as the illustrations can be prepared under Professor Wortman's direction.

*The President.*—It would seem that all philosophy has taken a step very much towards scientific demonstration, for as we look over the field we find that the religious men who are most thoroughly advanced in scientific studies, of whom there are a good many in England and some in this country, have gone quite as far in the acceptance of the doctrine of evolution as the professor has expressed himself this evening in his carefully chosen words and very moderate expressions. Indeed, a recent religious work of the highest type by George Matheson states that in no so dignified way could the creation of man be devised as by the one of evolution, which has been in recent years brought so prominently before us, and a careful examination of the best understood passages of Scripture that bear upon that subject and the comparison of them with the revelations of science show the fact that the two confirm each other most remarkably.

It is with much pleasure that we offer the thanks of the Institute to Professor Wortman for his very interesting discourse this evening, and hope that this is not the last time that we shall have the pleasure of seeing him among us.

Adjourned.

S. E. Davenport, D.D.S., M.D.S.,
*Editor The New York Institute of Stomatology.*

